# Prone positioning reduces frontal and hippocampal neuronal dysfunction in a murine model of ventilator-induced lung injury

**DOI:** 10.3389/fmed.2022.987202

**Published:** 2022-11-04

**Authors:** Nicklaus A. Sparrow, Gena Guidry, Faizan Anwar, Sonja Darwish, Scott A. Kelly, S. Ananth Karumanchi, Shouri Lahiri

**Affiliations:** ^1^Department of Neurology, Cedars-Sinai Medical Center, Los Angeles, CA, United States; ^2^Department of Medicine, Cedars-Sinai Medical Center, Los Angeles, CA, United States; ^3^Department Neurosurgery, Cedars-Sinai Medical Center, Los Angeles, CA, United States; ^4^Department of Biomedical Sciences, Cedars-Sinai Medical Center, Los Angeles, CA, United States

**Keywords:** ventilation-induced lung injury, interleukin-6 (IL-6), neuronal injury, frontal cortex, hippocampus, prone position, supine position, delirium

## Abstract

Prone positioning is an established treatment for severe acute lung injury conditions. Neuronal dysfunction frequently occurs with mechanical ventilation-induced acute lung injury (VILI) and clinically manifests as delirium. We previously reported a pathological role for systemic interleukin 6 (IL-6) in mediating neuronal injury. However, currently no studies have investigated the relationship between prone or supine positioning and IL-6 mediated neuronal dysfunction. Here, we hypothesize that prone positioning mitigates neuronal injury, via decreased IL-6, in a model of VILI. VILI was induced by subjecting C57BL/6J mice to high tidal volume (35 cc/kg) mechanical ventilation. Neuronal injury markers [cleaved caspase-3 (CC3), *c-fos*, heat shock protein 90 (Hsp90)] and inflammatory cytokines (IL-6, IL-1β, TNF-α) were measured in the frontal cortex and hippocampus. We found statistically significantly less neuronal injury (CC3, *c-Fos*, Hsp90) and inflammatory cytokines (IL-6, IL-1β, TNF-α) in the frontal cortex and hippocampus with prone compared to supine positioning (*p* < 0.001) despite no significant group differences in oxygen saturation or inflammatory infiltrates in the bronchoalveolar fluid (*p* > 0.05). Although there were no group differences in plasma IL-6 concentrations, there was significantly less cortical and hippocampal IL-6 in the prone position (*p* < 0.0001), indicating supine positioning may enhance brain susceptibility to systemic IL-6 during VILI via the IL-6 *trans*-signaling pathway. These findings call for future clinical studies to assess the relationship between prone positioning and delirium and for investigations into novel diagnostic or therapeutic paradigms to mitigate delirium by reducing expression of systemic and cerebral IL-6.

## Introduction

Prone positioning is a well-established treatment for patients with severe acute lung injury (1). Improving outcomes in this condition has taken on increased relevance during the current severe acute respiratory syndrome coronavirus 2 (SARS-CoV-2) pandemic (2). During acute lung injury, a change from supine to prone positioning is associated with minimized compression of the lungs by the heart and increased oxygenation resulting from improved ventilation–perfusion matching (3, 4). And, in cases of severe acute lung injury, prolonged prone-positioning has been shown to significantly decrease 28-day and 90-day mortality (5). In mechanical ventilation-induced acute lung injury (VILI), brain dysfunction of the frontal cortex and hippocampus frequently occurs and clinically manifests as delirium (6–8). Delirium concomitant with mechanical ventilation and severe acute lung injury contributes to an accelerated trajectory of long-term cognitive decline, increased mortality, and prolonged length of hospital stay (7, 8). We previously reported a pathological role for systemic interleukin 6 (IL-6) in mediating frontal and hippocampal neuronal injury in murine models of VILI and urinary tract infection (9, 10). In the VILI murine model, mechanically ventilated (35 cc/kg tidal volume) animals showed statistically significant increases in apoptotic markers and neuronal injury in the frontal cortex and hippocampus, but these markers were reduced following systemic IL-6 inhibition (10). In humans, at least one study has reported that prone positioning decreases systemic IL-6 compared to supine positioning in patients with ARDS due to community-acquired pneumonia (11). However, currently no studies in mice or humans have investigated the relationship between prone or supine positioning and IL-6 mediated neuronal dysfunction in the frontal cortex and hippocampus. In this study, we hypothesize that prone positioning mitigates neuronal injury in the frontal cortex and hippocampus, via decreased IL-6, in an animal model of VILI. Here, our objective is to evaluate the role of prone or supine positioning on systemic inflammation, neuroinflammation, and neuronal injury in response to VILI.

## Materials and methods

### Animals and power analysis

A total of 26 C57BL/6J mice (male and female), 6-7 months old (Jackson Laboratory, Bar Harbor, ME), were used in this study. Acute lung injury was induced in all mice and animals were assigned to either the prone (*n* = 6, males; *n* = 8, females) or supine (*n* = 4, males; *n* = 8, females) position during mechanical ventilation. As described previously (10), based on preliminary immunohistochemical analysis using cleaved caspase-3 (CC3), the greatest group SD and mean differences were 0.12 and 0.3, respectively. Using these values, a power analysis yielded greater than 90% power at the 0.05 significance level with *n* = 5/group. All experiments were conducted in accordance with Cedars-Sinai Medical Center’s Institutional Animal Care and Use Committee (IACUC) guidelines under an approved protocol and complied with current US law.

### Ventilation-induced acute lung injury model

Acute lung injury is achieved by subjecting mice to high tidal volume (35 cc/kg) mechanical ventilation, inducing stretch of alveolar walls. This can lead to cell deformation, endothelial and epithelial breaks, interstitial edema, and production of inflammatory infiltrates that can be measured in the bronchoalveolar lavage fluid (BALF) (10, 12). All procedures herein have been previously described (10, 13).

**FIGURE 1:**
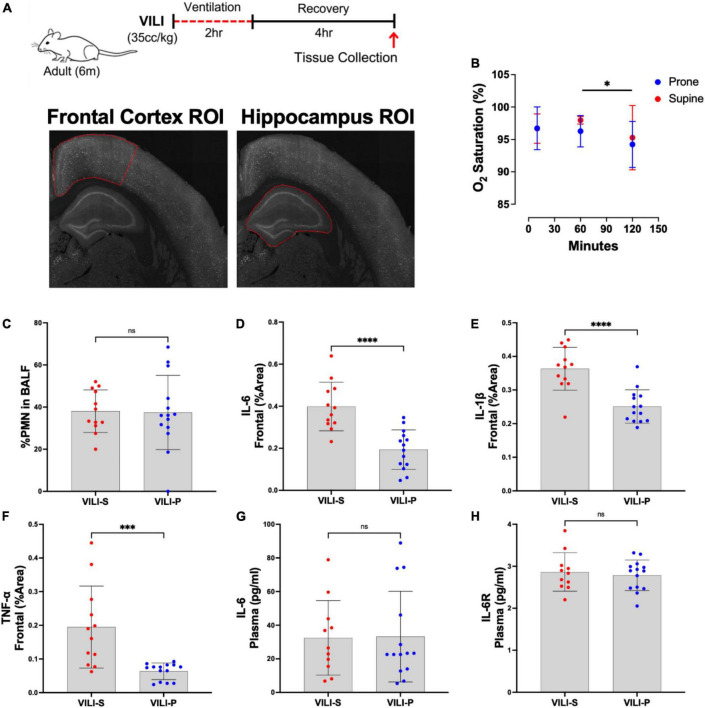
Prone position during acute ventilator induced lung injury (VILI) decreases neuronal inflammation in the frontal cortex. **(A)** Schematic of experimental timeline and representative regions of interest (ROIs) in the frontal cortex and hippocampus. **(B)** Oxygen saturation between prone and supine position did not significantly differ at any time point (*p* > 0.05). *Post hoc* tests revealed oxygen saturation at 120 mins (the end of the mechanical ventilation period) was significantly lower than at 60 min (*p* = 0.0328). **(C)** Quantification of lung inflammation measured by the percentage of polymorphonuclear cells (PMNs) in bronchoalveolar lavage fluid (BALF) was not significantly different between prone and supine groups, indicating that differential lung injury is not driving neuronal dysfunction. **(D–F)** Prone positioning significantly reduced cortical inflammatory cytokines (IL-6, IL-1β, TNF-α) as compared to supine positioned mice (*p* < 0.001). **(G,H)** Plasma IL-6 and IL-6 receptor (IL-6R) quantities did not differ between groups. Group sample sizes for all analyses were prone (*n* = 14) and supine (*n* = 12). Data are expressed as mean ± SD. *p < 0.05, ***p < 0.001, *⁣*⁣**p < 0.0001.

All mice were anesthetized with intraperitoneal injection of a mix of ketamine (Vedco Inc.) and dexmedetomidine (Pfizer) (75 mg/kg and 0.5 mg/kg, respectively), orotracheally intubated, and then mechanically ventilated using an Inspira volume-controlled small animal ventilator (Harvard Apparatus) and ambient, room air. Animals were mechanically ventilated in either supine or prone position and head position was kept constant in reference to the horizontal plane within each group. The mechanical ventilation parameters to induce acute lung injury were as follows: a tidal volume of 35cc/kg at a respiratory rate of 70 breaths per minute with zero positive end-expiratory pressure for a duration of 2 h (Figure 1A). A duration of 2 h of mechanical ventilation was utilized as previous studies indicate it is sufficient to induce VILI while mitigating attrition (10, 13, 14). The respiratory rate was set at 70 breaths/minute, which is appropriate for anesthetized mice who at baseline take 80-230 breaths/minute (15). Subcutaneous saline (0.5 mL) was administered to maintain hydration, and the eyes of mice were protected with a thin coat of Paralube (Dechra) immediately before intubation. During mechanical ventilation, the body temperature of mice was maintained using a 38°C heating pad (Hallowell EMC). Anesthesia was reversed with atipamezole (1 mg/kg in 100 μL of sterile water), and mice were allowed to recover in their cages on a heating pad for 4 h before euthanasia followed by tissue collection. As previously employed (10, 13), a 4-h recovery period was utilized so that observed brain histological changes were attributable to VILI and not confounded by immediate effects of anesthesia.

### Brain isolation and treatment

Following a 4 h recovery period after mechanical ventilation, mice were deeply anesthetized and perfused with room temperature phosphate buffered saline (PBS) with 0.5 mM ethylenediaminetetraacetic acid (EDTA) (10 mL). Perfusion with PBS with EDTA was utilized to ensure removal of blood from brain capillaries in preparation for histological analysis detailed below. Right brain hemispheres were collected and fixed by submerging in ice-cold PBS buffered 4% paraformaldehyde (Electron Microscopy Sciences) for 30 min, and then cryo-protected in 2% paraformaldehyde + 30% sucrose at 4°C for 48 h. Free-floating, 30-μm-thick coronal brain cryosections were prepared and stored at 4°C in PBS + 0.02% sodium azide until staining.

### Immunohistochemistry and microscopy

Brain sections were affixed to slides by air drying and subjected to heat induced epitope retrieval for 10min in antigen-retrieval solution (pH 6.0; Invitrogen) prior to permeabilization/blocking in 5% BSA + 0.25% Triton X-100 in PBS for 1 h at room temperature. Sections were then incubated at 4°C overnight with primary antibodies diluted in 1% BSA + 0.01% Triton X-100 in PBS (Ab Diluent). See Supplementary Table 1 for antibody information. After washing, sections were incubated with a combination of the appropriate secondary antibody (Alexa Fluor Plus conjugated; Invitrogen) diluted to 4 μg/mL in Ab Diluent for 1 h at room temperature. After washing, sections were incubated in 0.05% sudan black B in 70% ethanol for 10 min to reduce tissue autofluorescence. Sections were mounted using ProLong Glass with DAPI (Invitrogen). Negative controls were processed using the same protocol with the omission of the primary antibody to assess non-specific labeling. A Carl Zeiss AxioImager Z.2 epi-fluorescence microscope—equipped with standard filter sets/mercury arch lamp, an Apotome 2.0, and an Axiocam HRm camera—controlled by Zen Blue Pro (version 2.3) software was used to acquire and process images. Images of damage marker [CC3, *c-fos*, and heat shock protein 90 (Hsp90)] staining were acquired with a 10x objective (NA 0.3, Zeiss) as a 5 × 5 tiled image that encompassed both the neocortex and the hippocampus of each section. Images of cytokines [IL-6, interleukin-1β (IL-1β), and tumor necrosis factor α (TNF-α)] staining were acquired with the Apotome 2.0 and a 20x objective (NA 0.8, Zeiss) as a single field, 8 μm z-stacks (1-μm interval) and were analyzed and displayed as maximum intensity projections. All acquisition and display settings are the same between groups.

### Image and statistical analysis

Fiji (ImageJ v. 1.53c) software was used for image analysis and semi-quantitation. Prism 9.4.0 (GraphPad) was used for statistical analysis and analysis was performed by assessors blinded to group allocation. Three coronal sections containing the hippocampus and adjacent cortex were analyzed (one ventral, one mid, and one dorsal) per animal. For damage marker analysis, two different regions of interest (ROIs) were drawn on tiled images of sections: a ROI around the frontal neocortex beginning at the midline, or a ROI encompassing the entire hippocampus (both with an average area of 315 μm^2^) (Figure 1A). A threshold was set to exclude background pixels per ROI based on the pixel intensity histogram. The number of positive pixels was measured and then expressed as percent area of the ROI. For cytokine analysis, a single field z-stack projection from the frontal cortex was analyzed per section. Background pixels were excluded per field based on the pixel intensity histogram and the intensity of the remaining pixels was averaged to yield mean pixel intensity (mean PI) or used to calculate percent area. Values for each protein from the triplicate sections were averaged to yield one value per animal.

Statistically significant outliers were determined (ROUT method with a *Q* = 10%) and excluded, and unpaired Student’s *t*-tests were used to determine statistical significance between groups. A two-way repeated measures ANOVA (with *post hoc* tests) was utilized to determine the effects of position and time on oxygen saturation during mechanical ventilation. Regression analyses were utilized to examine putative causal relationships between variables. Data are presented as means ± SD and *p* < 0.05 was considered statistically significant.

## Results

There was no statistically significant difference in the age and weight of mice between groups (results not shown). During the recovery period following mechanical ventilation, all animals were active and ambulatory and showed no clinical evidence of shock. There was no significant difference between groups (supine vs. prone) in oxygen saturation at 10, 60, or 120 min after mechanical ventilation was initiated (Figure 1B). *Post hoc* analysis revealed that in both groups (prone and supine) oxygen saturation was significantly lower at 120 min (the end of the mechanical ventilation period) as compared to 60 min (*p* = 0.0328, Figure 1B). Additionally, there was no significant difference between groups in pulmonary inflammation as measured by increased percentage of polymorphonuclear cells (PMNs) in the BALF (Figure 1C). Histology indicated that lung injury was extensive, and severity did not differ between the position groups (Supplementary Figure 1A). Additionally, quantification using ELISA indicated there were no significant differences in BALF IL-6, IL-1β, or TNF-α between supine and prone positioned animals (Supplementary Figures 1B–D). Additionally, in a separate cohort of animals we assessed cerebral perfusion, quantified with MRI arterial spin labeling, between the supine and prone positions and observed no significant differences (Supplementary Figure 2). Thus, any group differences observed between the supine and prone groups are not attributable to differences in oxygen saturation, cerebral perfusion, or severity of VILI. In addition to the combined-sex analyses presented, we also examined sexes individually. Separate sex analysis yielded results consistent with combined analyses in both direction and magnitude of effect between the supine and prone groups. Consequently, in the combined analyses, one sex is not responsible for the overall group effects.

**FIGURE 2:**
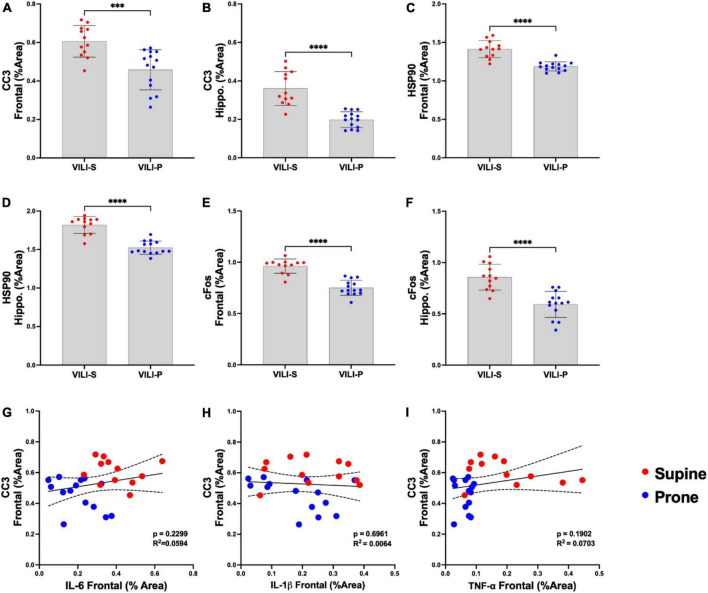
Prone position during acute ventilator induced lung injury (VILI) decreases neuronal injury and stress. **(A,B)** Frontal cortex and hippocampal cleaved caspase-3 (CC3), a known early marker of apoptosis, was statistically significantly reduced in a prone compared to supine position (*p* < 0.001). **(C,D)** In the prone position, heat shock protein 90 (Hsp90), a measure of cellular stress response, was significantly reduced in the frontal cortex and hippocampus (*p* < 0.0001) in comparison to that of supine positioning. **(E,F)** Prone positioning significantly reduced frontal cortex and hippocampal neuronal activity (C–FOS) compared to supine (*p* < 0.0001). **(G–I)** Regression analyses across both supine and prone groups demonstrated no statistically significant relationships between CC3 and inflammatory cytokines (IL-6, IL-1β, TNF-α) in the frontal cortex. Group sample sizes for all analyses were prone (*n* = 14) and supine (*n* = 12). Data are expressed as mean ± SD. ***p < 0.001, *⁣*⁣**p < 0.0001.

**FIGURE 3:**
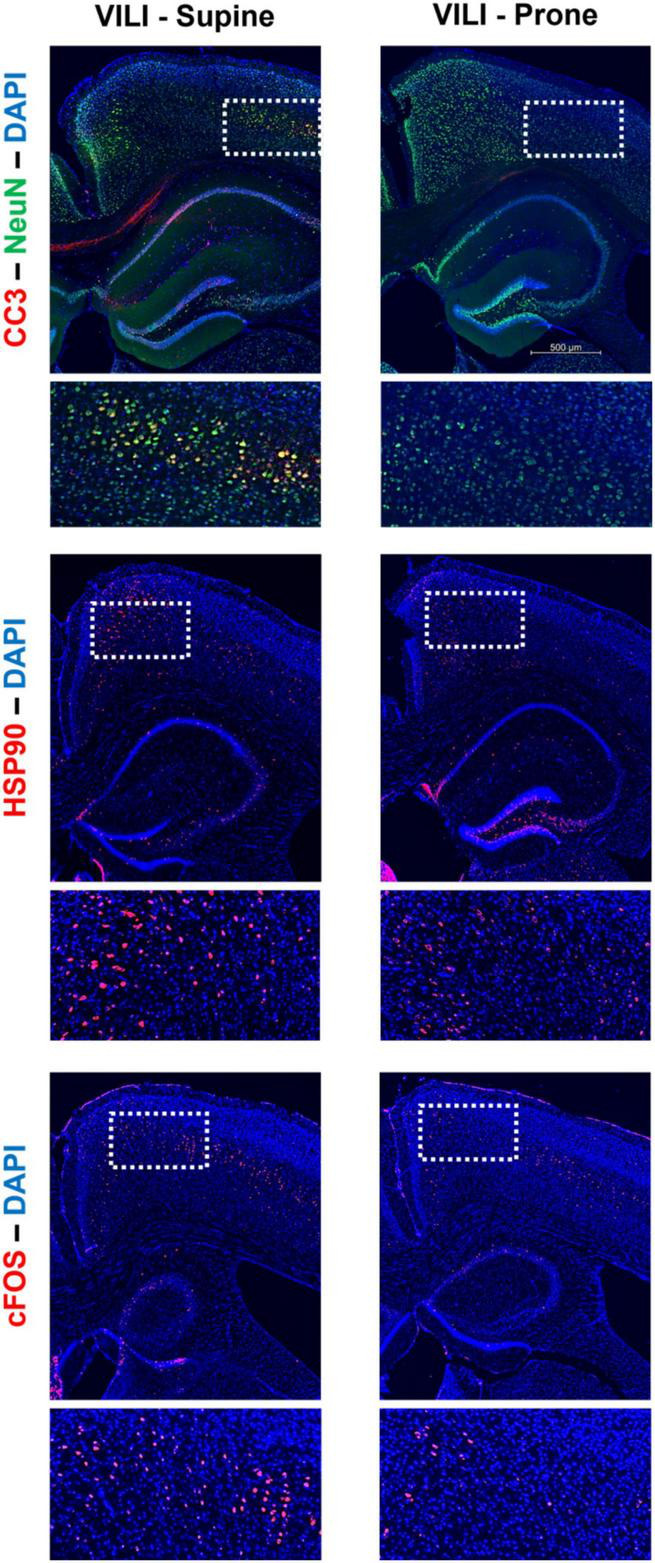
Characteristic staining of cortical cleaved caspase-3 (CC3), heat shock protein 90 (Hsp90), *c-Fos*, and neuronal nuclear protein (NeuN) in both supine and prone positioned mice.

**FIGURE 4:**
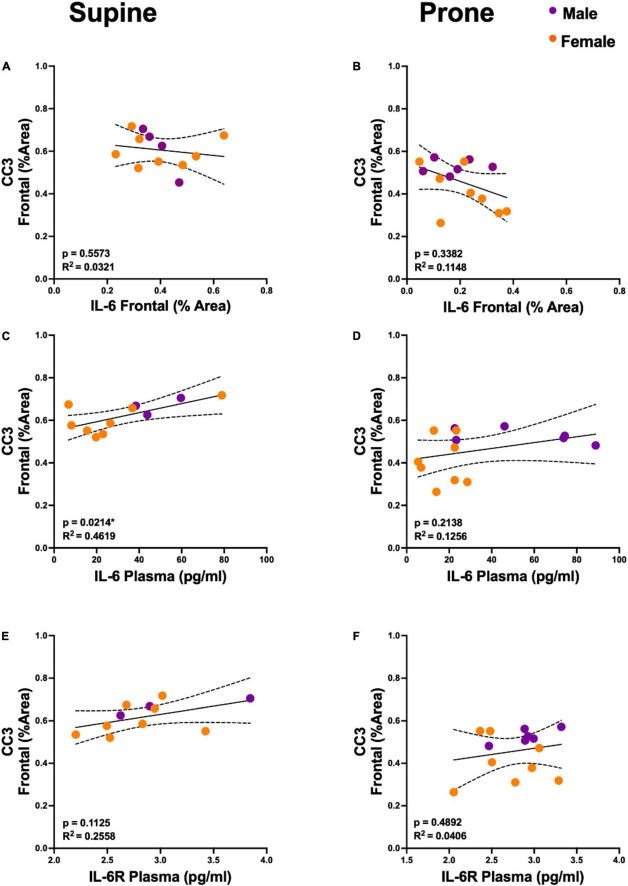
Plasma interleukin 6 (IL-6) is predictive of cortical cleaved caspase-3 (CC3) levels, but only in the supine position. **(A,B)** Regression analyses demonstrated no statistically significant relationship between CC3 and IL-6 in the frontal cortex. **(C,D**) Plasma IL-6 explained a significant proportion of variance in cortical CC3 in the supine group (R^2^ = 0.4619, *p* = 0.0214) but not in the prone positioned group (*R*^2^ = 0.1256, *p* = 0.2138). **(E,F)** There was no significant relationship between CC3 and plasma IL-6 receptor (IL-6R) concentration. Group sample sizes for all analyses were prone (*n* = 6, male; *n* = 8 females) and supine (*n* = 4, male; *n* = 8 females). Sexes are color coded, but partial regressions were not utilized as we observed the same directional group (supine vs. prone) differences in both males and females. Dotted lines represent 95% confidence intervals.

Figures 1, 2 demonstrate that prone positioning during acute VILI reduces cortical inflammation (IL-6, IL-1β, TNF-α) and decreases neuronal damage (CC3, Hsp90, *c-Fos*) in the frontal cortex and hippocampus. Interestingly, plasma IL-6 and IL-6R concentrations were not significantly altered by position (Figures 1G,H), potentially indicating enhanced brain susceptibility to IL-6 in the supine position. Figure 3 depicts representative cortical staining of CC3, HSP90, *c-Fos* and neuronal nuclear protein (NeuN) in both supine and prone positioned mice. Across both groups (supine and prone), no inflammatory cytokine (IL-6, IL-1β, TNF-α) explained a significant portion of the variability in CC3, a known early marker of apoptosis and neuronal injury within the cortex (Figures 2G–I) (16–18). Analyses within position groups showed no significant correlations between frontal IL-6 and frontal CC3 (Figures 4A,B). However, in the supine group alone, plasma IL-6 levels explained a statistically significant proportion of the variability in frontal cortex CC3 expression (*R*^2^ = 0.4619, *p* = 0.0214) (Figures 4C,D), while there were no significant correlations between plasma IL-6R and CC3 in either position (Figures 4E,F).

## Discussion

In this study, we found less neuronal injury and inflammatory cytokines in the frontal cortex and hippocampus with prone compared to supine positioning in a murine model of VILI. Additionally, we observed a significant positive relationship between plasma IL-6 and CC3 expression in the frontal cortex in the supine position but not in the prone. Collectively, these data suggest that prone positioning mitigates acute frontal and hippocampal brain dysfunction by lowering brain but not systemic IL-6 concentrations. In other words, supine positioning may enhance brain susceptibility to systemic IL-6 during VILI. These pre-clinical findings provide reassurance that the current strategy of prone positioning in acute lung injury does not aggravate acute frontal and hippocampal neuronal injury and accompanying delirium that frequently complicates recovery in patients with acute lung injury and, in fact, may ameliorate it. However, the exact underlying physiological mechanisms for the putative neuroprotective effect of prone positioning remain unclear. Future studies should be designed to evaluate whether exacerbation of neuronal injury in the supine position is due to impaired glymphatic clearance, as positioning has been previously shown to affect glymphatic function (19). Future studies should also be performed to assess whether differences in release of stress hormones between the two positions underlie the observed differences.

The IL-6 *trans*-signaling pathway refers to a pro-inflammatory process where peripheral IL-6 and soluble IL-6-receptor form a complex that can induce distal organ injury, such as in the brain (20–22). Prior studies have suggested that IL-6 *trans*-signaling may contribute to the pathogenesis of neurodegenerative conditions, such as dementia (22). The findings of this study suggest that the IL-6 *trans*-signaling pathway may be enhanced in the supine position and consequently mediate delirium-relevant neuronal injury induced by VILI in the supine but not the prone position. Supporting this hypothesis is the absence of a group effect in plasma IL-6 levels but higher cortical and hippocampal IL-6 in the supine position. As it is now known that delirium accelerates the trajectory of long-term dementia, the results of this study call for future research on the relationship between prone positioning, delirium, and dementia, in addition to further investigations into diagnostic or interventional paradigms that mitigate the neuropathology of delirium by lowering systemic or cerebral IL-6.

There are notable limitations of this study that justify consideration. The lack of behavioral functional data as a correlate to the histopathological changes is a limitation of this study. Unlike in patients, it is not possible to assess delirium in anesthetized mice undergoing mechanical ventilation. However, we have demonstrated neuronal injury, stress, and inflammation to the frontal cortex and hippocampus, anatomical structures that underlie the symptomatology of delirium (9). Further, we have previously shown that frontal and hippocampal CC3 expression correlates with delirium-like behavior (9). Additionally, inter-species differences in pulmonary anatomy, ventilation, and perfusion in mice compared to humans inherently limit the generalizability of the differences between supine and prone positions and their application to humans. However, at least one prior study in humans has found decreased systemic IL-6 with prone positioning, which may indirectly mitigate delirium-relevant neuropathology (11). Furthermore, although 35 cc/kg tidal volume is a previously published model of VILI (10, 13, 14), it is not a tidal volume typically used in patients and therefore the clinical relevance of our results may be limited. However, the lack of a precipitating cause of lung injury or concurrent systemic illness allows investigation of VILI-induced neuronal injury as an isolated variable, consistent with prior work (10, 13). Regardless, future studies of positioning effects in alternative models of lung injury are needed. In this study we did not ascertain the source of the systemic IL-6 – future studies are, thus, needed to trace the origins of cerebral IL-6 using RNA *in situ* hybridization of lung and brain tissue. Although cerebral perfusion, quantified using MRI arterial spin labeling, did not differ between the supine or prone positions (Supplementary Figure 2), future studies are indicated to evaluate the relationship between airway pressure or mechanical power of mechanical ventilation with brain hemodynamic changes. Finally, we recognize that there are advantages and disadvantages to any anesthetic regimen. In this study, both experimental groups received the same anesthetic regimen of dexmedetomidine and ketamine, thus any differences in neuronal injury between the groups should not be explained by anesthetic effects alone. Furthermore, dexmedetomidine is known to have among the most favorable effects on delirium of any anesthetic while clinical studies have demonstrated attenuation of postoperative delirium following treatment with ketamine (23–25).

In summary, this study provides first evidence that prone positioned mice subjected to VILI have less neuronal injury in the frontal cortex and hippocampus compared to supine positioned mice with VILI. Additional studies are needed to evaluate the mechanisms behind the enhanced susceptibility to neuronal injury in the supine position following VILI. Although we found no positional differences in cerebral perfusion, future studies should specifically evaluate additional hemodynamic variables as potential factors that may explain the positional differences. These findings call for future clinical studies to assess the relationship between prone positioning and delirium and for investigations into novel diagnostic or therapeutic paradigms to mitigate delirium by reducing expression of systemic and cerebral IL-6.

## Data availability statement

The raw data supporting the conclusions of this article will be made available by the authors, without undue reservation.

## Ethics statement

This animal study was reviewed and approved by Cedars-Sinai Medical Center’s Institutional Animal Care and Use Committee (IACUC).

## Author contributions

NS and SL conceived the study and were responsible for the experimental design. NS, GG, FA, and SD performed the experiments. NS, GG, FA, and SL analyzed the data. NS, GG, SKe, SKa, and SL interpreted the data and drafted the manuscript. All authors reviewed, edited, and approved the final version of the article.

## Supplementary material

Supplementary Figure 1 Position (supine vs. prone) during acute ventilator induced lung injury (VILI) does not differentially affect severity of injury or bronchoalveolar lavage fluid (BALF) cytokine levels. **(A)** Both supine and prone ventilated mice show evidence of acute lung injury (VILI). Low (I & II) and high (III & IV) magnification of representative micrographs of H&E-stained lung cryosections (8 μm) collected from supine (I & III) and prone (II & IV) VILI mice. A single bronchus (Br), as well as multiple alveoli (Av) can be seen within the low magnification micrographs. Regardless of the ventilation position, lung tissues demonstrated characteristic VILI histological findings. Specifically, there is increased thickness and eosinic staining of alveolar walls, in addition to increased abundance of polymorphonuclear cells, mainly neutrophils (indicated by arrows). These findings are consistent with lung inflammation and injury typically observed in VILI. **(B)** interleukin-6 (IL-6), **(C)** interleukin-1 beta (IL-1β), and **(D)** tumor necrosis factor-alpha (TNF-α) BALF concentrations (pg/mL), quantified using ELISA, were not significantly affected by position (supine vs. prone).

Supplementary Figure 2 Position (supine vs. prone) during acute ventilator induced lung injury (VILI) does not differentially affect cerebral perfusion (ml/100g*min). Cerebral perfusion, as measured using arterial spin labeling magnetic resonance imaging, in supine (*n* = 3) compared to prone (*n* = 2) positioned VILI mice.

## References

[1] Guérin C Albert RK Beitler J Gattinoni L Jaber S Marini JJ Prone position in ARDS patients: why, when, how and for whom. *Intensive Care Med.* (2020) 46:2385–96. 10.1007/s00134-020-06306-w 33169218 PMC7652705

[2] Coppo A Bellani G Winterton D Di Pierro M Soria A Faverio P Feasibility and physiological effects of prone positioning in non-intubated patients with acute respiratory failure due to covid-19 (PRON-COVID): a prospective cohort study. *Lancet Respir Med.* (2020) 8:765–74. 32569585 10.1016/S2213-2600(20)30268-X PMC7304954

[3] Albert RK Hubmayr RD. The prone position eliminates compression of the lungs by the heart. *Am J Respir Crit Care Med.* (2000) 161:1660–5. 10.1164/ajrccm.161.5.9901037 10806172

[4] Richter T Bellani G Scott Harris R Vidal Melo MF Winkler T Venegas JG Effect of prone position on regional shunt, aeration, and perfusion in experimental acute lung injury. *Am J Respir Crit Care Med.* (2005) 172:480–7. 10.1164/rccm.200501-004OC 15901611 PMC2718529

[5] Guérin C Reignier J Richard JC Beuret P Gacouin A Boulain T Prone positioning in severe acute respiratory distress syndrome. *N Engl J Med.* (2013) 368:2159–68. 23688302 10.1056/NEJMoa1214103

[6] Albaiceta GM Brochard L Dos Santos CC Fernández R Georgopoulos D Girard T The central nervous system during lung injury and mechanical ventilation: a narrative review. *Br J Anaesth.* (2021) 127:648–59. 10.1016/j.bja.2021.05.038 34340836

[7] Ely EW Shintani A Truman B Speroff T Gordon SM Harrell FE Jr Delirium as a predictor of mortality in mechanically ventilated patients in the intensive care unit. *JAMA.* (2004) 291:1753–62. 10.1001/jama.291.14.1753 15082703

[8] Sasannejad C Ely EW Lahiri S. Long-term cognitive impairment after acute respiratory distress syndrome: a review of clinical impact and pathophysiological mechanisms. *Crit Care.* (2019) 23:352. 10.1186/s13054-019-2626-z 31718695 PMC6852966

[9] Rashid MH Sparrow NA Anwar F Guidry G Covarrubias AE Pang H Interleukin-6 mediates delirium-like phenotypes in a murine model of urinary tract infection. *J Neuroinflam.* (2021) 18:247. 10.1186/s12974-021-02304-x 34711238 PMC8554965

[10] Sparrow NA Anwar F Covarrubias AE Rajput PS Rashid MH Nisson PL IL-6 inhibition reduces neuronal injury in a murine model of ventilator-induced lung injury. *Am J Respir Cell Mol Biol.* (2021) 65:403–12. 10.1165/rcmb.2021-0072OC 34014798 PMC8525200

[11] Chan MC Hsu JY Liu HH Lee YL Pong SC Chang LY Effects of prone position on inflammatory markers in patients with ARDS due to community-acquired pneumonia. *J Formos Med Assoc.* (2007) 106:708–16. 10.1016/S0929-6646(08)60032-7 17908660

[12] Matute-Bello G Frevert CW Martin TR. Animal models of acute lung injury. *Am J Physiol Lung Cell Mol Physiol.* (2008) 295:L379–99. 10.1152/ajplung.00010.2008 18621912 PMC2536793

[13] Anwar F Sparrow NA Rashid MH Guidry G Gezalian MM Ley EJ Systemic interleukin-6 inhibition ameliorates acute neuropsychiatric phenotypes in a murine model of acute lung injury. *Crit Care.* (2022) 26:274. 10.1186/s13054-022-04159-x 36100846 PMC9469063

[14] Wilson MR Choudhury S Goddard ME O’Dea KP Nicholson AG Takata M. High tidal volume upregulates intrapulmonary cytokines in an in vivo mouse model of ventilator-induced lung injury. *J Appl Physiol.* (2003) 95:1385–93. 10.1152/japplphysiol.00213.2003 12807894

[15] Schwarte LA Zuurbier CJ Ince C. Mechanical ventilation of mice. *Basic Res Cardiol.* (2000) 95:510–20. 10.1007/s003950070029 11192374 PMC7102075

[16] Burguillos MA Deierborg T Kavanagh E Persson A Hajji N Garcia-Quintanilla A Caspase signalling controls microglia activation and neurotoxicity. *Nature.* (2011) 472:319–24. 10.1038/nature09788 21389984

[17] Venero JL Burguillos MA Joseph B. Caspases playing in the field of neuroinflammation: old and new players. *Dev Neurosci.* (2013) 35:88–101. 10.1159/000346155 23445938

[18] D’Amelio M Cavallucci V Middei S Marchetti C Pacioni S Ferri A Caspase-3 triggers early synaptic dysfunction in a mouse model of Alzheimer’s disease. *Nat Neurosci.* (2011) 14:69–76. 10.1038/nn.2709 21151119

[19] Lee H Xie L Yu M Kang H Feng T Deane R The effect of body posture on brain glymphatic transport. *J Neurosci.* (2015) 35:11034–44. 10.1523/JNEUROSCI.1625-15.2015 26245965 PMC4524974

[20] Campbell IL Erta M Lim SL Frausto R May U Rose-John S Trans-signaling is a dominant mechanism for the pathogenic actions of interleukin-6 in the brain. *J Neurosci.* (2014) 34:2503–13. 10.1523/JNEUROSCI.2830-13.2014 24523541 PMC6802757

[21] Narazaki M Yasukawa K Saito T Ohsugi Y Fukui H Koishihara Y Soluble forms of the interleukin-6 signal-transducing receptor component gp130 in human serum possessing a potential to inhibit signals through membrane-anchored gp130. *Blood.* (1993) 82:1120–6. 10.1182/blood.V82.4.1120.1120 8353278

[22] Wang WY Tan MS Yu JT Tan L. Role of pro-inflammatory cytokines released from microglia in Alzheimer’s disease. *Ann Transl Med.* (2015) 3:136. 10.3978/j.issn.2305-5839.2015.03.49 PMC4486922 26207229

[23] Hudetz JA Patterson KM Iqbal Z Gandhi SD Byrne AJ Hudetz AG Ketamine attenuates delirium after cardiac surgery with cardiopulmonary bypass. *J Cardiothorac Vasc Anesth.* (2009) 23:651–7. 19231245 10.1053/j.jvca.2008.12.021

[24] Hovaguimian F Tschopp C Beck-Schimmer B Puhan M. Intraoperative ketamine administration to prevent delirium or postoperative cognitive dysfunction: a systematic review and meta-analysis. *Acta Anaesthesiol Scand.* (2018) 62:1182–93. 10.1111/aas.13168 29947091

[25] Ng KT Shubash CJ Chong JS. The effect of dexmedetomidine on delirium and agitation in patients in intensive care: systematic review and meta-analysis with trial sequential analysis. *Anaesthesia.* (2019) 74:380–92. 10.1111/anae.14472 30367689

