# Supplementary material for: Prone positioning reduces frontal and hippocampal neuronal dysfunction in a murine model of ventilator-induced lung injury
Source: Front Med (Lausanne). 2022 Nov 4;9:987202. doi: 10.3389/fmed.2022.987202 (PMC9674088; doi:10.3389/fmed.2022.987202)
Supplement: Supplementary file 1 [file Data_Sheet_1.PDF]

SUPPLEMENTAL TABLE 1. Antibodies used for immunohistochemistry.

Antigen	Dilution	Vendor	Catalog #
c-fos	500	Santa Cruz	SC-52
CC3	800	Cell Sig.	9664
HSP90	300	Life Tech.	37-9400
IL-1β	150	Cell Sig.	12242S
IL-6	150	Life Tech.	AMC0864
TNF-α	150	Cell Sig.	11948S
