# Supplementary figures and images for: Prone positioning reduces frontal and hippocampal neuronal dysfunction in a murine model of ventilator-induced lung injury
Source: Front Med (Lausanne). 2022 Nov 4;9:987202. doi: 10.3389/fmed.2022.987202 (PMC9674088; doi:10.3389/fmed.2022.987202)

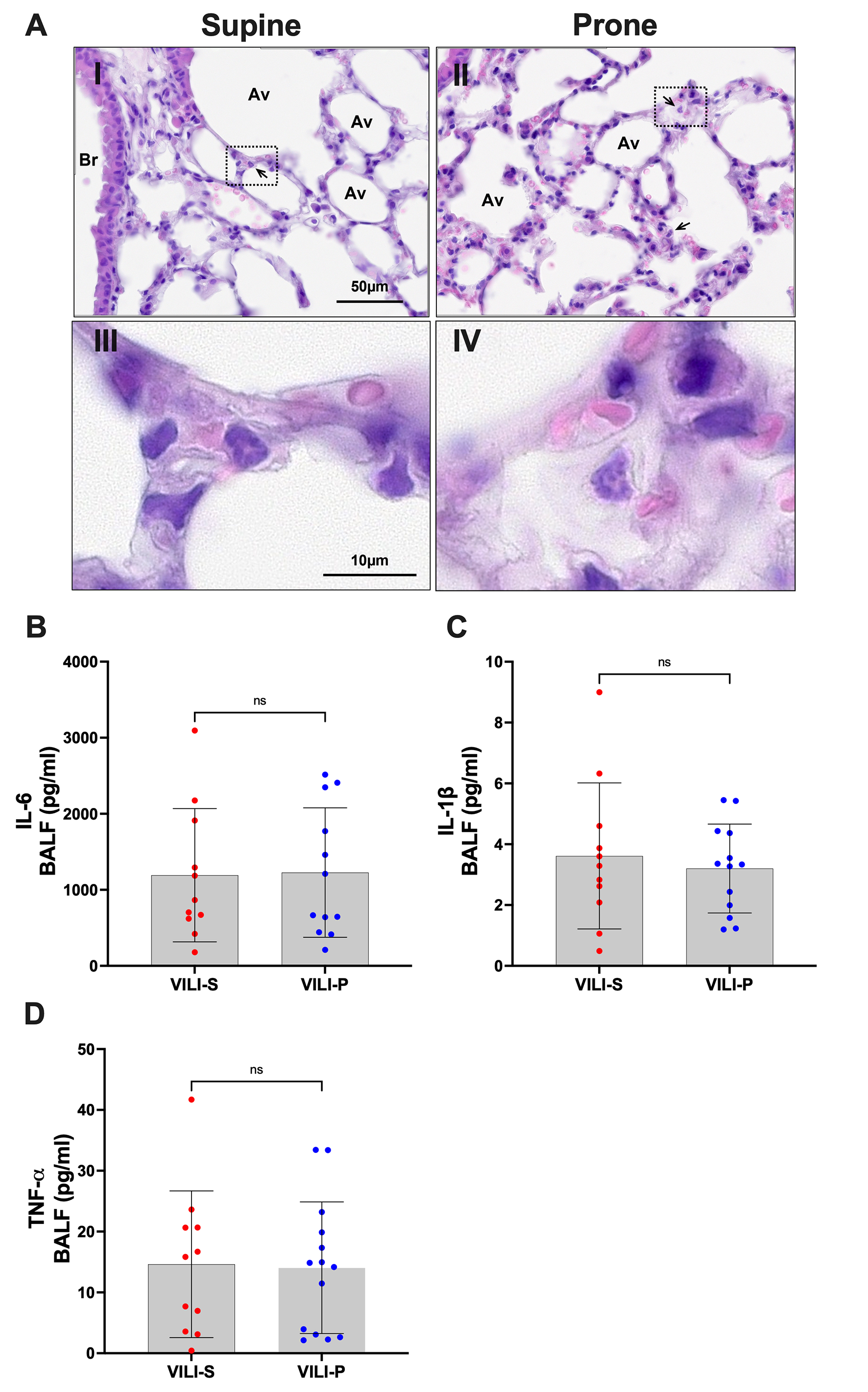

Supplement: Supplementary Figure 1 — Position (supine vs. prone) during acute ventilator induced lung injury (VILI) does not differentially affect severity of injury or bronchoalveolar lavage fluid (BALF) cytokine levels. (A) Both supine and prone ventilated mice show evidence of acute lung injury (VILI). Low (I & II) and high (III & IV) magnification of representative micrographs of H&E-stained lung cryosections (8 μm) collected from supine (I & III) and prone (II & IV) VILI mice. A single bronchus (Br), as well as multiple alveoli (Av) can be seen within the low magnification micrographs. Regardless of the ventilation position, lung tissues demonstrated characteristic VILI histological findings. Specifically, there is increased thickness and eosinic staining of alveolar walls, in addition to increased abundance of polymorphonuclear cells, mainly neutrophils (indicated by arrows). These findings are consistent with lung inflammation and injury typically observed in VILI. (B) interleukin-6 (IL-6), (C) interleukin-1 beta (IL-1β), and (D) tumor necrosis factor-alpha (TNF-α) BALF concentrations (pg/mL), quantified using ELISA, were not significantly affected by position (supine vs. prone). [file Image_1.TIFF]

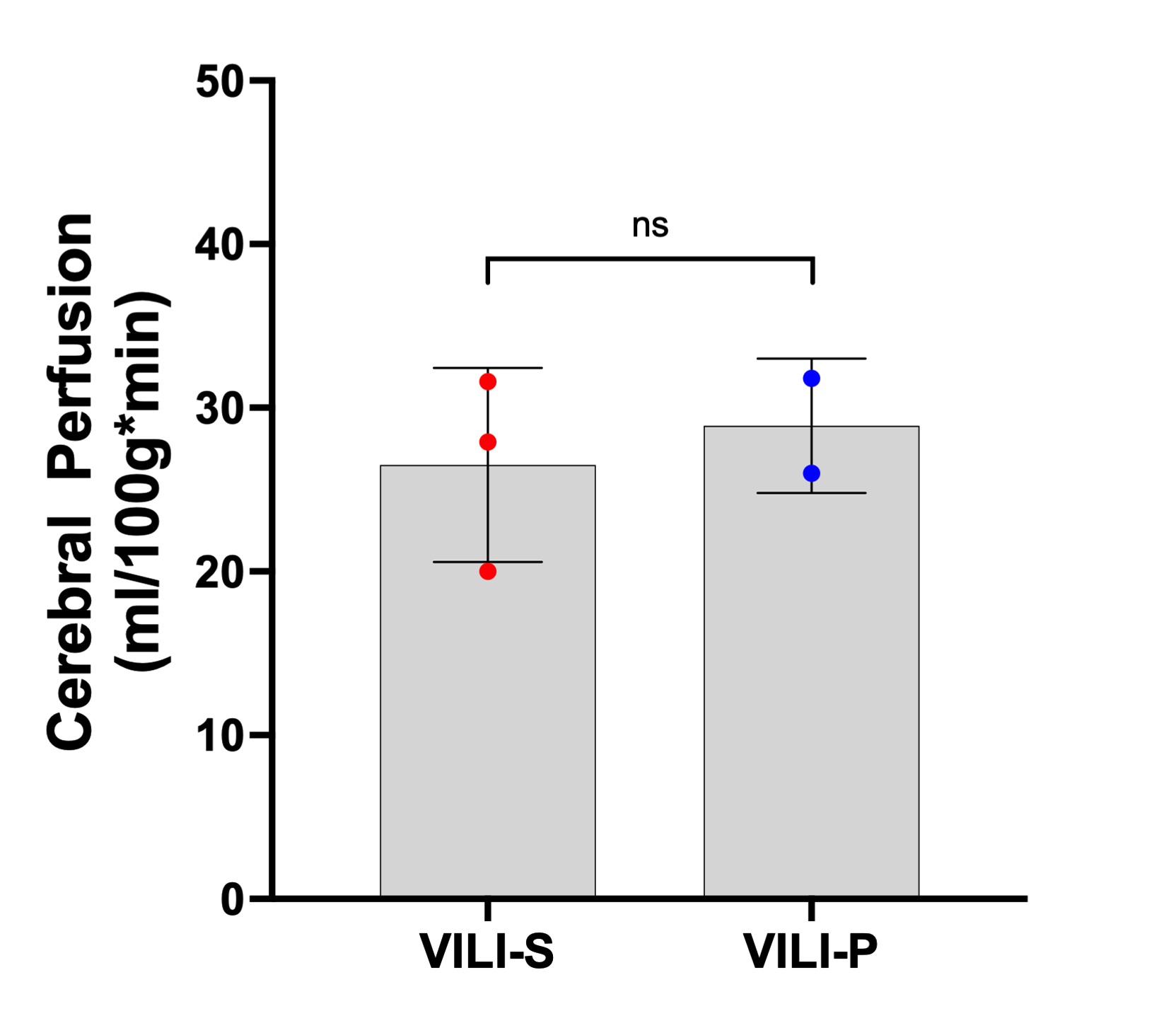

Supplement: Supplementary Figure 2 — Position (supine vs. prone) during acute ventilator induced lung injury (VILI) does not differentially affect cerebral perfusion (ml/100g*min). Cerebral perfusion, as measured using arterial spin labeling magnetic resonance imaging, in supine (n = 3) compared to prone (n = 2) positioned VILI mice. [file Image_2.TIFF]
